# Notes and News

**Published:** 1893-09-30

**Authors:** 


### NOTES AND NEWS.

COLLECTED AND COMMUNICATED.

THE foundation-stone of a new infirmary for the Guisborough Workhouse was laid on September 19th. The estimated cost of the new building is £3,235.

THE sixteenth course of lectures given at the Sanitary Institute, Parkes Museum, will begin on Tuesday next, October 3rd, at eight p.m., when the lecturer will be Sir Douglas Galton, and the subject "Ventilation, Warming, and Lighting." A preliminary lecture is announced for the 29th of this month, on "Elementary Physics and Chemistry."

SPECIAL interest attaches to the distribution of prizes at St. Thomas's Hospital on October 3rd, as it will be the occasion for the presentation of a testimonial to Dr. Bristowe from past and present colleagues and students of St. Thomas's. The subscriptions have reached the sum of nearly £400, and Dr. Bristowe is founding and endowing with a large part of the money a medal to be named after himself, and awarded annually for proficiency in pathology to a student of the hospital. Dr. Bristowe's portrait has been painted by Miss Beatrice Bristowe for the subscribers.

AT a meeting of the executive committee of the Hospital Saturday Fund, held last week under the presidency of the treasurer, Mr. H. N. Hamilton Hoare, to consider the steps to be taken in regard to the alleged defalcations in the Norwood collection boxes, alluded to in another column, it was decided to place the whole facts before the Public Prosecutor, when it may be hoped that the matter will be probed to the bottom. The street collections are unpopular, and if it can be proved that the contents of the boxes are tampered with, the end will not be far off. Neither the giving public nor the lady collectors will tolerate street collections in future, should it be found that their labours and offerings are utilised by rascals for their own benefit. We hope the Public Prosecutor will take up the matter, as it is one which properly belongs to his department.

THE Scotch form of oath-taking by holding up the right hand seems likely to become gradually more customary, thanks to the increasingly frequent refusal of members of the medical profession and others to follow the insanitary and highly objectionable practice of "kissing the book." There can be no doubt in the minds of reasonable people that such a custom may be a fruitful source of infection, and it cannot be too widely known that all persons "having authority to administer oaths are bound to administer the Scotch form of oath to any person who desires to be so sworn, without asking any questions."

THE following directions for the prevention of cholera have been distributed throughout the district of St. John and St. Mary's, Westminster, by the Medical Officer of Health: "No accumulation of refuse of any kind should be permitted. All drinking water should be boiled, filtering not being sufficiently reliable. Care should also be taken that the vessels used for drinking purposes are kept clean. All water cisterns should be thoroughly cleansed, and should be emptied and cleansed at frequent intervals. No fruit, meat, &c., should be eaten if in the slightest degree stale. Eating unripe or over-ripe fruit or ice creams should be avoided. The walls of areas, backyards, &c., should be thoroughly limewashed, and drains should be frequently flushed with carbolic acid, which will be supplied gratuitously upon application at the Town Hall."

THE Birmingham Hospital Saturday Fund is going ahead with a vengeance. Having established a convalescent home for men, it has now determined to raise a debenture loan of £10,000, repayable in twenty years, bearing interest from 3 to 4 per cent., payable half-yearly, to enable the committee to purchase and provide a suitable house and furniture for a Women's Convalescent Home. The debentures are to be in amounts of £10, or multiples of £10, and the Hospital Saturday authorities believe that there are numbers of working men who will gladly avail themselves of this method of investment. We shall watch this experiment with interest, although the principle it represents is not free from danger, and we are not prepared to advocate an extension of the system by other similar organisations, at any rate for the present.

NUNEATON may be justly proud of the efforts which have resulted in the opening of a new Cottage Hospital there last week. The want of such an institution has been sadly felt for some time, indeed no less than fifteen years ago a scheme was initiated for its erection, but for various reasons the plans fell through. A more successful fortune has attended a District Nursing Institution, since started, and subscriptions flowed in so well that the Cottage Hospital scheme was again brought forward, and the building was commenced some ten months ago. The hospital contains seventeen beds, and is fitted with all modern improvements and conveniences. With it will be amalgamated the existing District Nursing Association, which has done much good work, and is deservedly appreciated. It is to be hoped that this new departure may meet with equal success, and that those who have given so liberally towards it may be ably supported by the entire population of Nuneaton and the neighbourhood.

